# Mammary epithelial morphogenesis in 3D combinatorial microenvironments

**DOI:** 10.1038/s41598-020-78432-w

**Published:** 2020-12-10

**Authors:** Raphaelle Luisier, Mehmet Girgin, Matthias P. Lutolf, Adrian Ranga

**Affiliations:** 1 Genomics and health informatics group, Idiap Research Institute, 1920 Martigny, Switzerland; 2 Laboratory of Stem Cell Bioengineering, Institute of Bioengineering, School of Life Sciences and School of Engineering, Ecole Polytechnique Fédérale de Lausanne, 1015 Lausanne, Switzerland; 3 Institute of Chemical Sciences and Engineering, School of Basic Sciences, Ecole Polytechnique Fédérale de Lausanne, 1015 Lausanne, Switzerland; 4 Laboratory of Bioengineering and Morphogenesis, Biomechanics Section, Department of Mechanical Engineering, KU Leuven, 3001 Leuven, Belgium

**Keywords:** Mammary stem cells, High-throughput screening, Biomaterials

## Abstract

Human mammary epithelial cells can proliferate and reorganize into polarized multi-cellular constructs in-vitro, thereby functioning as an important model system in recapitulating key steps of in-vivo morphogenesis. Current approaches to constructing such three-dimensional mimics of the in-vivo microenvironment have involved the use of complex and ill-defined naturally derived matrices, whose properties are difficult to manipulate independently, and which have therefore limited our ability to understand the extrinsic regulation of morphogenesis. Here, we employ an automated, high-throughput approach to array modular building blocks of synthetic components, and develop a systematic approach to analyze colonies resulting from these varied microenvironmental combinations. This methodology allows us to systematically map the relationship between microenvironmental properties and ensuing morphogenetic phenotypes. Our analysis reveals that apico-basal polarity of mammary epithelial cells occurs within a narrow range of matrix stiffness, and that phenotypic homogeneity is favored in matrices which are insensitive to MMP-mediated degradation. Furthermore, combinations of extracellular proteins in the matrix finely tune the morphology of the mammary colonies, suggesting that subtle disregulations of the microenvironment may play a significant role in pathological disease states. This approach, which leverages the combinatorial possibilities of modular synthetic artificial extracellular matrices with an automated technology platform, demonstrates how morphogenesis can be assessed systematically in 3D, and provides new insights into mammary epithelial multicellularity.

## Introduction

The form and function of epithelial tissues is defined by the three-dimensional microenvironment in which they reside^1^. In-vitro models which can recapitulate aspects of normal morphogenesis and the progression to epithelial tumors have been critical in understanding the molecular mechanisms underlying these processes. In the context of epithelial morphogenesis, as well as of tissue-level mimics of morphogenesis termed organoids, naturally derived matrices such as collagen and Matrigel have been widely utilized. While these materials allow for remarkable and spontaneous recapitulation of three-dimensional architecture, their utility in understanding the role of the extrinsic microenvironment is severely limited due to the inability to decouple their structural and signaling properties, as well as by their complex composition and significant lot-to-lot variability. In recent years, synthetic approaches to materials engineering have provided a set of artificial extracellular matrices (aECM) which have highly reproducible and manipulable material properties, as well as a biologically neutral background onto which bioactive elements can be selectively grafted^2^. These materials allow for modular and independent exploration of various physicochemical properties and have most often been used to explore the role of the microenvironment on single cell^3–5^ or multicellular cancer-derived assemblies^6–8^.

More recently, these aECMs have begun to be exploited in the context of epithelial morphogenesis, where a minimal synthetic microenvironment was able to recapitulate features of intestinal morphogenesis^9–11^ as well as early neuroepithelial morphogenesis^12,13^ in a manner analogous to that observed in Matrigel. The role of stiffness, degradability and adhesion ligands has also been extensively investigated in the context of morphogenesis of MDCK cysts, a commonly used model of renal morphogenesis which recapitulates hallmarks of kidney distal tubule and collecting ducts, in the form of characteristic acini^10^. As extensive and informative as these studies have been, they also underscore the notion that the available parameter space available for biological exploration with modular hydrogel systems is no longer limited by the material capabilities but rather by the time constraints of conventional manual experimentation. To address this limitation and to broaden and systematize the use of synthetic artificial microenvironments, we have developed a high-throughput 3D microarray platform which has allowed for the investigation of systems-level regulation of embryonic stem cell self-renewal in murine embryonic stem cells^14^ as well as the optimization of such microenvironments for somatic cell reprogramming^15^.

While these model systems develop into multicellular colonies, they do not exhibit elements of morphogenesis. We have employed our 3D microarray platform to explore the role of the microenvironment in early multicellular neural differentiation^13^ and found that the development of neural tube organoids, as has been reported for cerebral organoids^16^, exhibited rapid emergence of apico-basal polarity, indicating that this morphogenetic process may be conserved across early embryonic stem cell-derived developmental fates.

Here, we thought to employ this 3D aECM microarray framework to systematically explore the role of the microenvironment in regulating the emergence of adult multicellular epithelial morphogenesis. To do so, we made use of MCF10A cells, an immortalized mammary epithelial line, which, like MDCK, function as a commonly used model of epithelial morphogenesis and have additional relevance due to their human identity. Indeed, MCF10A cells polarize when encapsulated in Matrigel (or on a Matrigel substrate with dilute Matrigel in the medium)^17^ to form acini structures that resemble terminal mammary buds. In order to study morphogenesis in a rapid and automated manner, we also develop an approach to deal with high content 3D imaging data, which, combined with statistical analysis techniques, allows us to understand the morphogenetic response of epithelial cells to the microenvironment in a systematic way.

## Results

### Building a customized materials library targeted to epithelial morphogenesis

**Figure 1:**
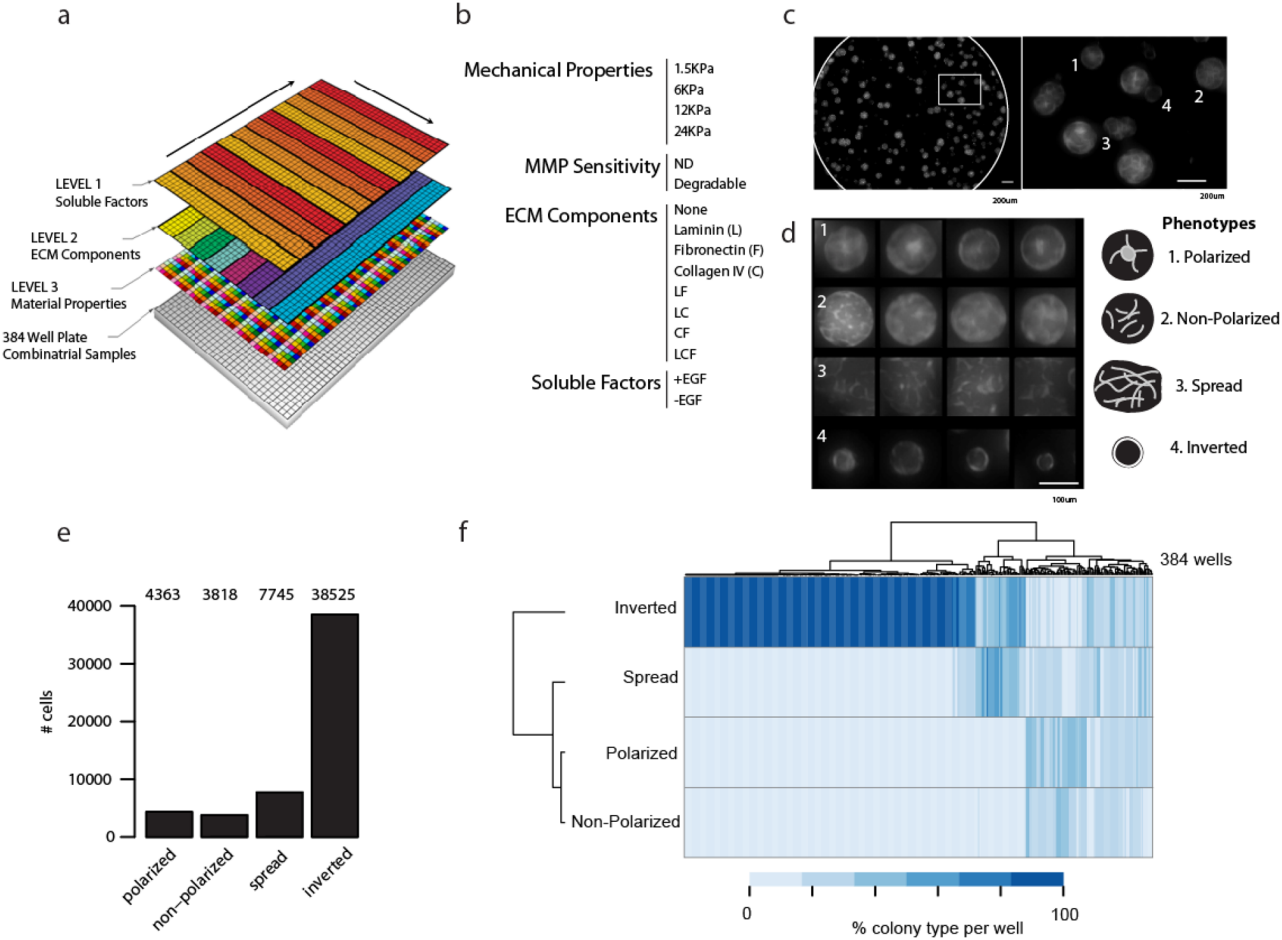
Artificial extracellular matrix screening platform for mammary epithelial cells. Multiple levels of microenvironmental control are constructed with an automatic liquid handling robot (**a**). List of components which are combinatorially arrayed in this screen (**b**). Whole-well 3D imaging is performed to reveal four phenotypic classes (**c**,**d**) and heterogeneous cellular phenotypes within each well. Quantification of the number of phenotypically distinct colonies across the 384 wells (**e**). Hierarchical clustering analysis of the frequency of the four phenotypes across the 384 wells (**f**). Scale bar 200 µm in c and 100 μm in d.

In this study, we make use of a robust and versatile robotic liquid handling platform to array a combination of hydrogel-based microenvironments. The first step in this process is the choice of material components and concentrations in the starting library. Here, we employ synthetic polyethylene glycol (PEG) precursors as neutral backbone components, whose physicochemical properties can be tuned in a range of orthogonal ways, which we term the “materials matrix” (Material Properties in Fig. 1a). Additional levels of microenvironmental modulation accessible via our robotic approach include control over extracellular matrix (ECM) components (ECM components in Fig. 1a) and as well as medium composition overlaying the matrix (Soluble Factors in Fig. 1a). We hypothesize that optimal morphogenesis could occur in the range of stiffness material previously reported for normal non-pathologic mammary epithelial tissue^18^, and that matrix degradability, or sensitivity to MMP degradation, could be a key component of the microenvironment. As such a matrix stiffness range of 0.5 to 8 kPa was modulated here by modifying the polymer content (1% to 3% w/v), whereas the sensitivity of the matrix to MMPs was modulated by incorporating peptide substrates with differential sensitivity to proteolytic degradation at the PEG termini. In addition to the physical aspects of the microenvironment involved in maintaining dimensionality and matrix mechanics, we also hypothesize that signaling from extracellular matrix (ECM) components could play a key role in affecting morphogenesis. Our choice of ECM materials to include in this library reflected our interest in deconstructing Matrigel, the gold standard matrix in morphogenesis studies, which is largely composed of laminin and collagen IV. We also included fibronectin, which is not considered a significant element in the basement membrane and has not been especially implicated in mammary morphogenesis, but has important roles in enhancing adhesion, growth and differentiation in contexts such as wound healing, cancer and fibrosis^19^. To examine whether combinations of such ECM played important synergistic or antagonistic roles when presented in combination, we also specified in our experimental design all possible combinations of these three key ECM components. Finally, as an additional level of modulation, we included EGF in the form of a soluble factor: while this growth factor is necessary for proliferation of MCF10A cells in monolayer and in reported 3D cultures thus far, we thought to assess whether synthetic matrices could replace such exogenously added factors. In summary we tested in triplicate a combination of 128 different culture conditions with varying matrix stiffnesses, degradability, and ECM component signaling in presence or absence of the proliferative growth factor EGF (n = 384 wells; Fig. 1b).

### Assessing heterogeneous phenotypes

After seven days of incubation, the cell culture arrays were fixed and stained with phalloidin to reveal the cytoskeletal organization of the colonies. Segmentation identified 56,892 colonies across the 384 wells with a median number of 159 ± 47 colonies per well (Fig. 1c,d,e).

While it is commonly assumed that treatment conditions lead to homogenous phenotypes reflecting common biological mechanisms, subpopulations exhibiting distinct phenotypes can be found within the same well^20^. Indeed, visual inspection of the segmented colonies revealed heterogeneous mixtures of phenotypes populating most wells (Fig. 1c). These could be grouped into four phenotypically distinct categories: (1) apico-basally “polarized” colonies, exhibiting a round colony with a central actin belt, (2) “inverted” colonies with actin cytoskeletal bundles concentrated on the edges, (3) “non-polarized” colonies with round morphology but unpolarized cytoskeleton, and (4) “spread” colonies with diffuse actin fibers, exhibiting a larger, less organized morphology (Fig. 1c,d).

### Automated colony sorting into four phenotypically distinct categories

We next aimed to gain a better understanding of the sizes and distributions of the visually identified four types of colonies. Given the complexity of biological phenotypes which characterized our mixture of colonies, we selected the open-source Cell Profiler Analyst software to interactively classify the 56,892 colonies on the basis of their morphometric similarity with the visually identified four groups^21^. Importantly, single metrics of object classification such as colony intensity or size could not be used for the complex morphogenetic traits we thought to assess here. 67% of the colonies were classified using a Random Forest model as inverted, 13% as spread, 7.7% as polarized and 6.7% as non-polarized (n_inverted_ = 38,525, n_spread_ = 7,745, n_polarized_ = 4,364 and n_non-polarized_ = 3818; Fig. 1e). Co-occurrence of polarized and non-polarized colonies in identical wells, as revealed by hierarchical clustering of the categories using the frequency profile of the 384 wells, suggested that these two groups are closely related and induced in similar culture conditions, while inverted and polarized/non-polarized were determined to be largely mutually exclusive (Fig. 1f).

### Assessing phenotypes using Singular Value Decomposition

**Figure 2:**
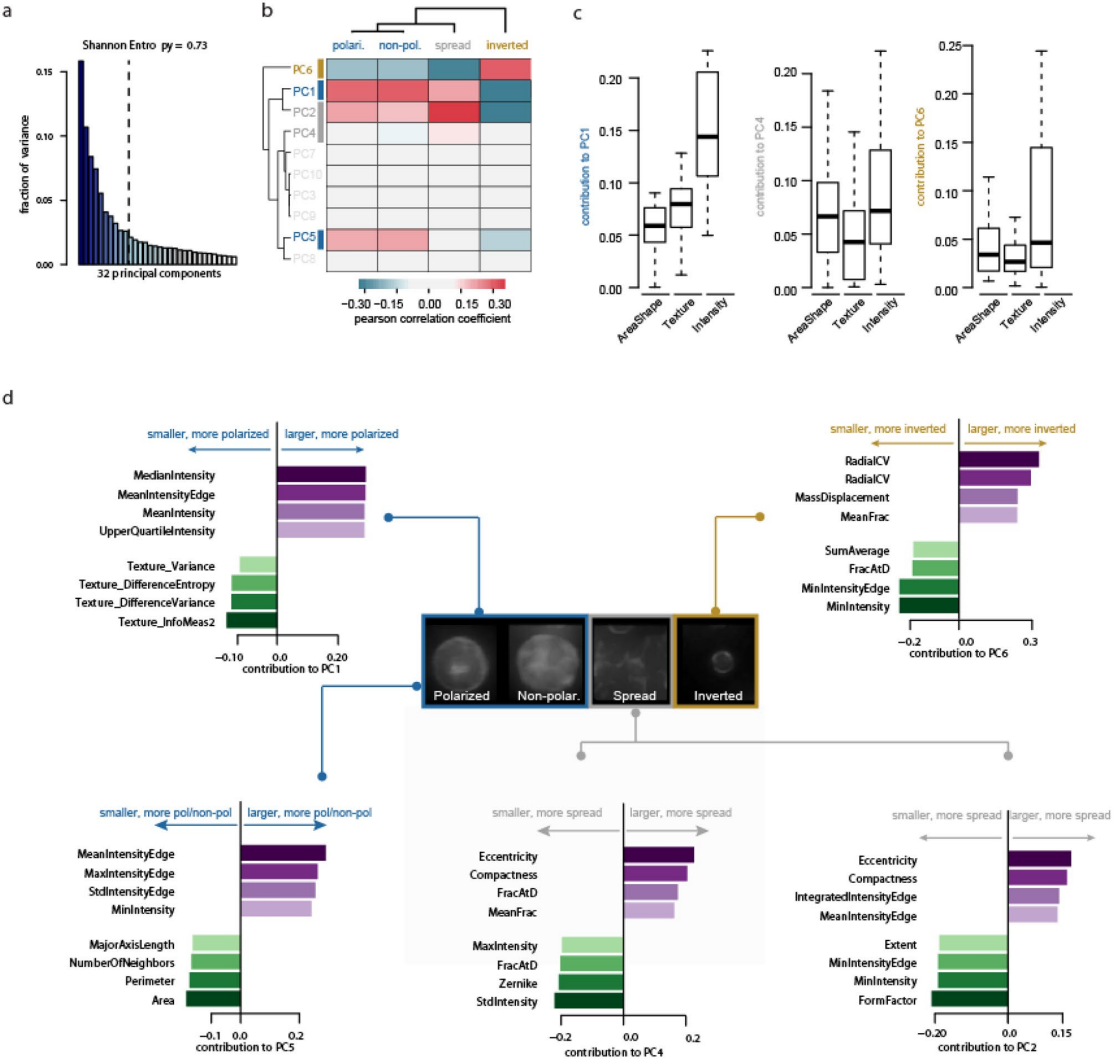
Unsupervised analysis of the most frequent phenotypes. The top 10 principal components (PCs) obtained from the singular value decomposition analysis of the 102 measurements across 56,892 colonies captures 73% of the variance in the data and are used for downstream analysis (**a**). Correlation analysis between the ten first left singular vectors and the frequency of visually identified phenotypes within each well (**b**). Distribution of the absolute contribution of the 102 measurements grouped in three categories (areashape, texture and intensity) to selected PCs (**c**). Identification of the measurements contributing most positively and most negatively to the selected PCs as indicative imaging features having most and least influence on selected PCs (**d**).

We then sought to test whether the four distinct phenotypes visually identified naturally emerged from high-content microscopy data using dimensionality reduction and unsupervised analysis. We performed Singular Value Decomposition (SVD) analysis of the scaled high-content microscopy data (102 measurements across 56,892 colonies) to extract the most informative colony morphological profiles. While the first 31 principal components of the SVD analysis captured 90% of the variance in the data (Fig. 2a), we selected the first ten principal components for downstream analysis given that they captured more than 73% of the signal (indicated by the dashed bar). We then tested the association between the visually identified colony phenotype categories and the ten first principal components using Pearson correlation scores (PCCs) of the individual left singular vectors and the frequency of cells in each category. We found association between PC1 (15% of variance), PC2 (10% of variance), and PC6 (4% of variance) with *inverted* colony frequency, confirming the dominance of *inverted* phenotype in the data (Fig. 2b). Although the distinction between *polarized/non-polarized* colony frequency was not captured by any component, the correlation between PC5 (5.5% of variance) with both *polarized* and *non-polarized* colony frequencies supports the existence of a large group composed of these two phenotypes (Fig. 2b). Finally *spread* colony frequency only slightly correlated with PC4 (7.4% of variance).

We then tested whether different types of measurements (area-shape, texture or intensity) were contributing differently to the PCs that underlie these different groupings, i.e. PC1, PC4, and PC6 (Fig. 2c). We found that *polarized/non-polarized* phenotype is best captured by a combination of texture and intensity-based measurements, as indicated by large contribution of these to PC1. Extracting the four top positive and negative contributing measurements to PC1 further indicated that *polarized/non-polarized* are best characterised by large intensity of the colony in particular at the edges, and low variance of the texture (Fig. 2d). *Spread* phenotype is best captured by a combination of area-shape and intensity, indicated by large contribution of these to PC4, while texture was not contributing to this phenotype classification (Fig. 2c). Large eccentricity and compactness (i.e. irregular object) and low intensity are indeed characteristics of this phenotype (Fig. 2d). Both PC1 and PC5 correlate with *polarized/non-polarized* phenotype, however only PC1 slightly correlates with *spread* phenotype*.* Further comparing the top contributors to PC1 with those of PC5 further revealed that *polarized/non-polarized* phenotype is characterised by smaller area and perimeter compared to spread phenotype (Fig. 2d). *Inverted* phenotype is best captured by intensity-based measurements, as indicated by large contributions of these into PC6 (Fig. 2c), and is characterised by large coefficient of variation of intensity (RadialCV) as well as low min intensity (Fig. 2d). Altogether this analysis supports the existence of three broad phenotypes (*polarized/non-polarized, spread and inverted*) that are captured by distinct measurements, and suggests that the morphological differences between *polarized* and *non-polarized* are too subtle to be captured by this analysis.

### Phenotype-based global factor analysis

**Figure 3:**
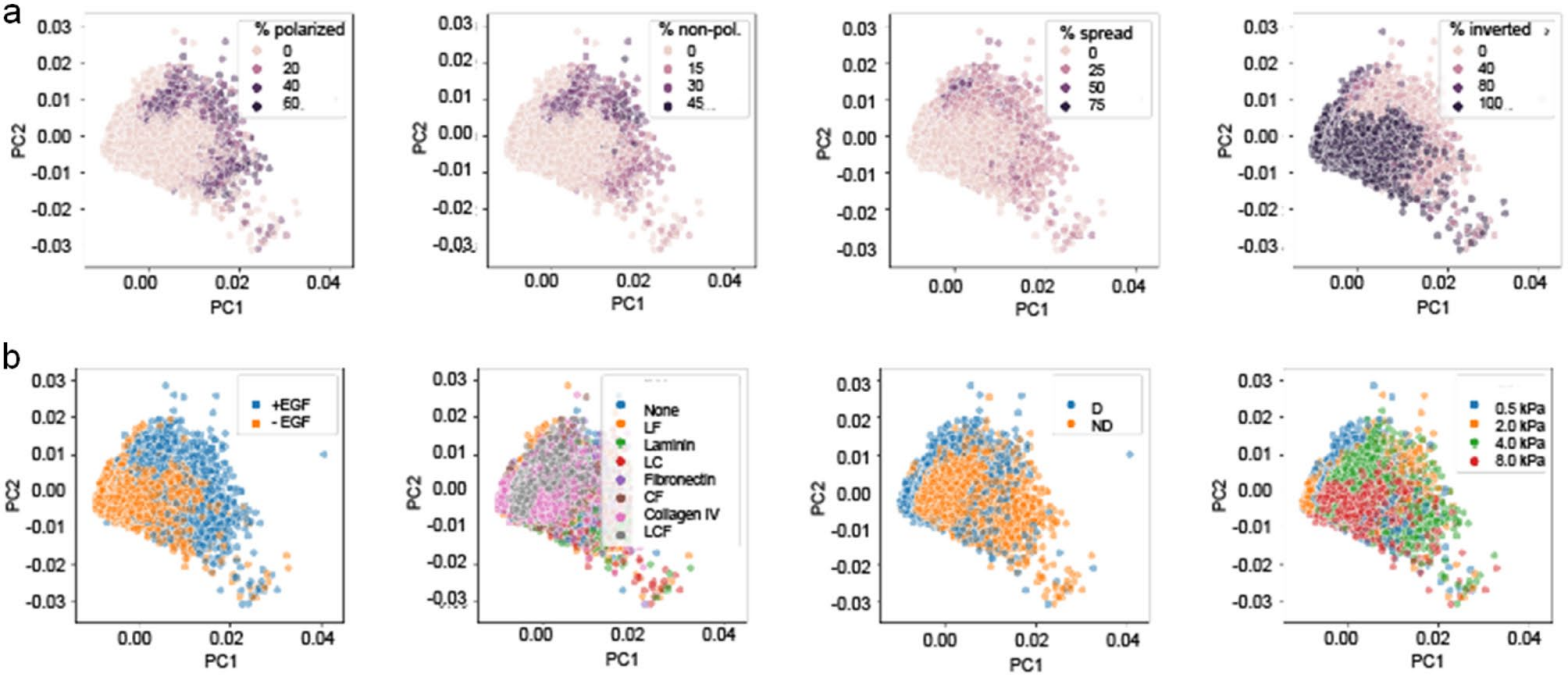
Feature mapping on principal components 1 and 2. Visualization of phenotype frequency (**a**) and microenvironmental factors on principal components 1 and 2 (**b**).

**Figure 4:**
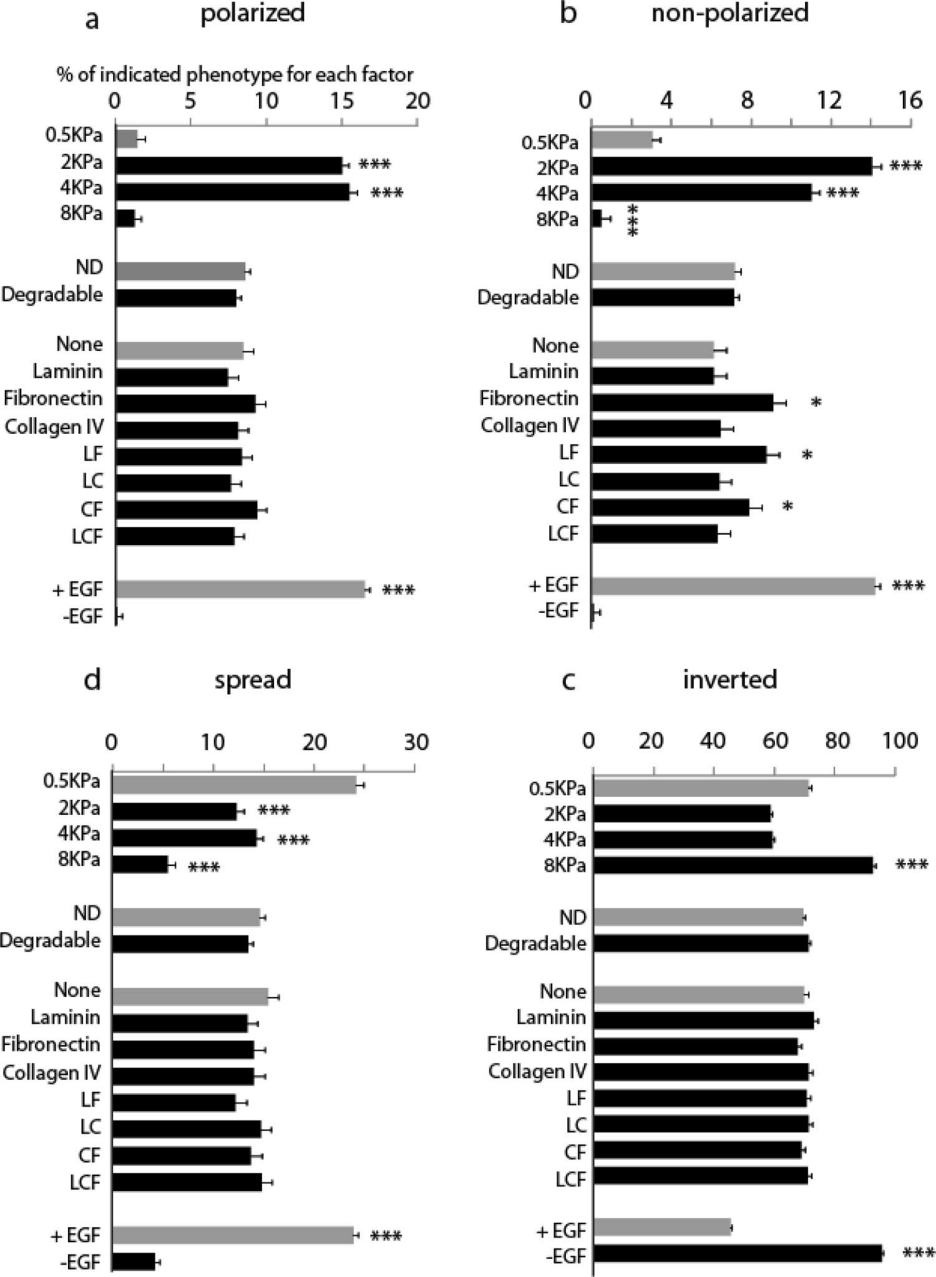
Global factor analysis for mammary epithelial colony phenotype. A GLM model is developed for each phenotype, which provides a characteristic microenvironmental signature for (**a**) polarized, (**b**) non-polarized, (**c**) spread and (**d**) inverted colonies. LF: laminin + fibronectin, LC: laminin + collagen IV, *CF* collagen IV + fibronectin, *LCF* laminin + collagen IV + fibronectin. The numbers on the x-axis indicate the average proportion of each phenotype within each well, for the given factor within the category. Statistical analysis identifies the significance of the change with respect to the condition in light gray for every category. Error bars represent s.e.m. All pairwise differences were computed using the Tukey–Kramer method. ***P < 0.001, **P < 0.01, *P < 0.05.

We next aimed to understand the systematic microenvironmental conditions underlying the development of phenotypically distinct mammary epithelial colonies. We first visualised the 56,892 colonies onto the two first PCs iteratively color-coding them with the frequency of phenotype present in the well they originate from, further confirming 1) the overlap between *polarized* and *non-polarized phenotypes* and 2) the homogenous grouping of the *spread* phenotype in PC1 and PC2 (Fig. 3a)*.* Further color-coding the colonies with the different conditions of the four categories of treatment (presence or absence of EFG, varying ECM component signaling, degradability and varying matrix stiffnesses; Fig. 3b) revealed that the absence of EGF as well as large matrix stiffness correlate with high frequency of *inverted* phenotype. Furthermore this visual analysis suggests that the degradability of the matrix leads to enrichment of *spread* phenotype as opposed to *polarized* and *non-polarized phenotypes.* Using a generalized linear model (GLM)^14^ we next statistically analyzed the relationship between the proportions of visually identified four colonies with each unique microenvironmental condition (Fig. 4). The lack of EGF treatment and/or a high matrix stiffness (8 kPa) emerged as the most influential microenvironmental conditions, leading to ~ 100% of inverted colonies per well. This indicates that high matrix stiffness and/or lack of growth factor are sufficient conditions to prevent apico-basal morphogenesis, at least at the relatively early time points studied here. Globally the GLM analysis revealed a dominant role of the mechanical properties of the matrix to the establishment of each of the four different phenotypes: an intermediate matrix stiffness regime (between 2 and 4 kPa) was a requirement for the establishment of both *polarized* and *non-polarized* colonies, with < 5% of either colonies in wells of either 0.5 or 8 kPa matrix stiffness; *non-polarized* colonies were slightly favored in 2 kPa condition compared to 4 kPa which was not the case of *polarized* which grow equally well across these the two matrix stiffnesses; the *spread* phenotype was favored in lowest matrix stiffness.

The MMP sensitivity of the matrix did not lead to significant changes in any of the colony phenotypes in this averaged global analysis. Similarly the global contribution of ECM matrix incorporation was not a predominant effector of *polarized*, *spread* or *inverted* phenotypes. However, the incorporation of fibronectin (either alone or together with collagen IV or laminin) significantly enhanced the appearance of *non-polarized* colonies, at the expense of *polarized* colonies. This result suggests that given the right matrix for normal epithelial development, notably an intermediate matrix stiffness and the presence of EGF, fibronectin would inhibit the initiation and establishment of apico-basal polarity.

**Figure 5:**
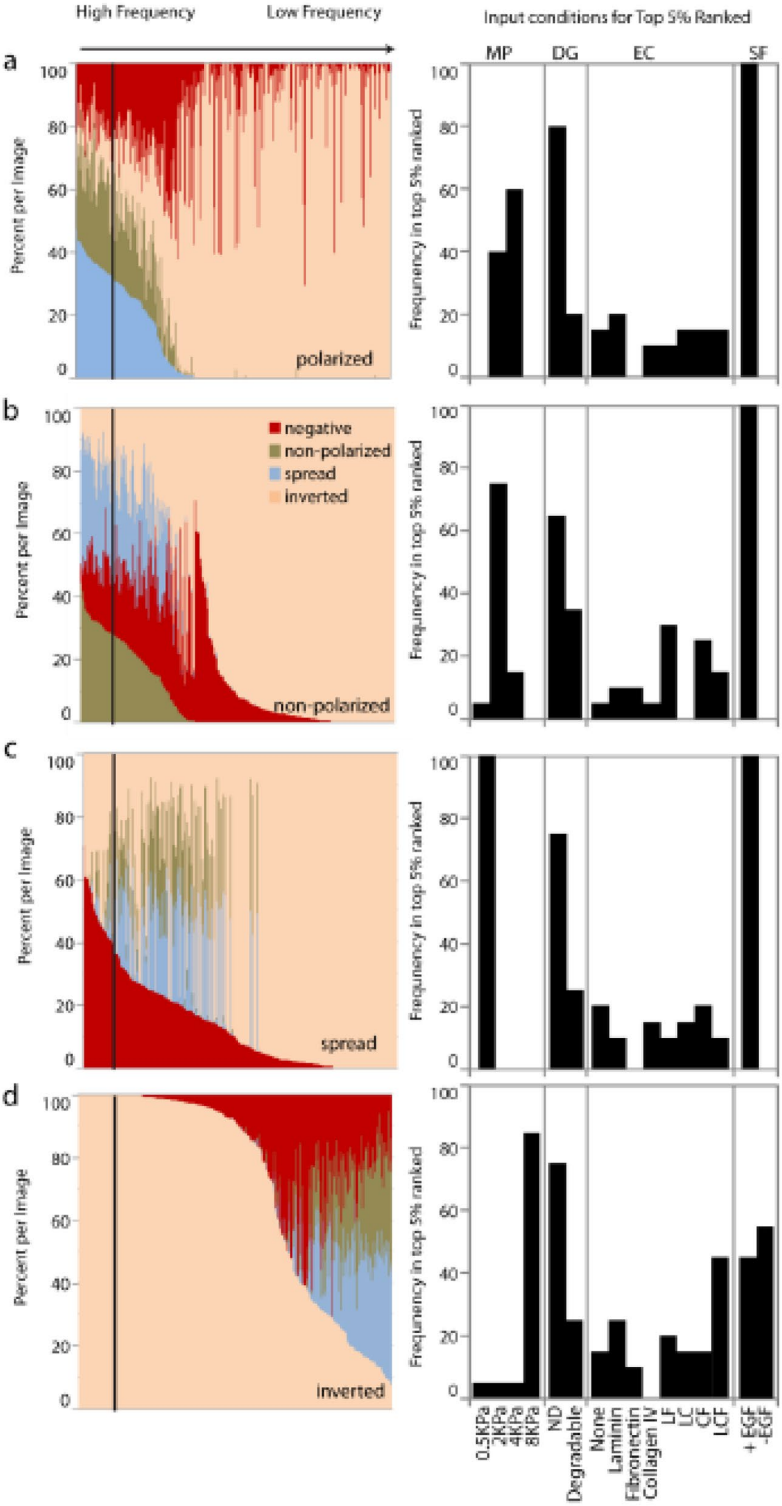
Ranked phenotype analysis. Wells are ranked by frequency of each phenotype, with top 5% ranked wells for each phenotype mapped to initial microenvironmental conditions.

As most wells contain varying mixtures of phenotypes, to identify the optimal ECM conditions that favor particularly high expression of each morphologies, we selected for each phenotype the top 5% wells with highest corresponding frequency and mapped back their corresponding input microenvironmental conditions (Fig. 5). We visualized the relative frequency of all four phenotypes in each well, reordering the wells in decreasing order frequency for each individual phenotype. While this approach validated our previous GLM-based finding, i.e. that *polarized* and *non-polarized* colonies are favored by intermediate matrix stiffness, it also shed light on subtle but important variations of ECM in favoring either the *polarized* or *non-polarized* phenotypes that are overlooked using global analyses. While the top 5% of *polarized* and *non-polarized* colonies are in similar materials matrix space (mechanical properties and degradability categories), fibronectin is completely absent in the optimal conditions for *polarized* colonies, while combinations of ECMs including fibronectin (LF, CF, LCF), but not LC favor the *non-polarized* phenotype. Similarly, a combination of all ECM conditions (LCF) leads to particularly high frequencies of inverted colonies, suggesting that multiple overlapping ECM cues, in conjunction with high stiffness, lead to mixed signaling and aberrant morphogenesis. This analysis also allowed us to reveal negative regulators of mammary epithelial morphogenesis: for example, high frequencies of *polarized* and *spread* colonies are not evidenced in fibronectin conditions while inverted colonies are not evidenced in collagen IV conditions. In addition, this analysis reveals an additional role for matrix degradability: while with GLM analysis there appears to be no global difference between phenotypes in degradable and non-degradable conditions, the top 5% of each phenotype appears in non-degradable matrix, suggesting that that a non-degradable matrix promotes homogeneous phenotype, while a degradable matrix allows for more phenotypic diversity. Finally, the discrete analysis of highly enriched phenotypes allowed us to unequivocally discount the possibility that conditions of optimal matrix properties could support the proliferation and polarity of MCF10A in the absence of exogenously added EGF. While a global analysis allows us to understand the regulatory space of the complete population for each phenotype, the use of frequency maps therefore permits a clearer delineation of individual conditions for optimal emergence, in frequency and homogeneity, of such phenotypes.

## Discussion

In this study, we show how a versatile 3D microenvironmental screening approach could be implemented to study mammary epithelial morphogenesis. We use here both supervised and unsupervised colony classification approaches to identify and quantify 3D colony phenotypes present across the 384 different culture conditions. This approach, combined with both systems-level GLM analysis as well as discrete, frequency maps-based identification of highly enriched phenotypes, allows us to identify global patterns of microenvironmental regulation, as well as more specific conditions leading to high-frequency and high-homogeneity expression of epithelial colony phenotypes. This unbiased systems-level analysis of morphogenesis in 3D allows us to rapidly identify a landscape of mammary epithelial morphogenesis regulators. Importantly, the phenotype nomenclatures “polar”, “non-polar”, “spread” and “inverted” proposed here are designations which may be further confirmed with specific polarity markers such as GM130 and podxl. Nonetheless, they serve here as useful, albeit reductionist designations in a high-throughput, single-marker context. Our analysis also identifies principal components of multiple image features associated with these four phenotypes, and imaging features which are most closely linked to these principal components are identified.

Our results delineate a relatively narrow matrix stiffness range between 2 and 4 kPa in which MCF10A polarization can occur. This is in line with a previous study using synthetic matrices, which found that polarization of MDCK cysts was only initiated around 4kPa^10^. However, important differences are noted here with respect to MDCK cysts: at low stiffness MCF10A tend to spread, whereas MDCKs tend to exhibit inverted polarity. Conversely, it is at higher stiffness that MCF10A cysts exhibit inverted polarity, whereas MDCK cysts in such stiff matrices were growth arrested and did not polarize^10^.

Our results show a previously unreported “inverted” polarity phenotype for MCF10A cysts occurring mainly in synthetic matrices of high stiffness, and which is distinct from the more invasive colonies reported at later times in stiffened matrices^22^. Notably, the F-actin staining of our non-polarized phenotype corresponds to the actin distribution of MCF10A cysts grown in hypoxic conditions, which have been suggested to resemble a cancer-like phenotype in primary human breast epithelial cells^23^.

The results of this study also reveal a novel role for matrix degradability in the context of MCF10A morphogenesis. While previous experiments with MDCK cells in synthetic matrices suggested that some measure of MMP-degradation of the PEG network was essential for normal epithelial morphogenesis^10^, our global analysis find that MMP sensitivity does not affect phenotype, but conversely, an analysis of top conditions for each phenotype suggests that phenotype homogeneity is favored in non-degradable matrices.

Another important conclusion from this study is the relevance of fibronectin in disrupting polarity. While the fibronectin-derived adhesion peptide RGD has been previously used and allows for polarity in MDCK^10^, this may not be equivalent for full length fibronectin Fn). Indeed, this is supported by data on conditional knockout of Fn in the mouse epithelium, which shows that Fn deletion causes retardation in outgrowth and branching of the ductal tree in 5-week old mice, and that mammary glands consisting of Fn-deficient epithelial cells fail to undergo normal lobular differentiation during pregnancy^24^ .

The study of epithelial morphogenesis is instrumental to the understanding of disease processes where epithelial polarity is disrupted. Recent work has shown how large-scale image-based analysis can be used to characterize the response of intestinal organoids to drug libraries^25^. Here we show how a systematic, automated approach can be used to investigate the influence of the microenvironment on mammary epithelial phenotype, and demonstrate how synthetic matrices can modulate both normative and pathological cellular responses. This methodology is relatively easy to implement, and is therefore amenable to larger data sets, including, importantly, dynamic time-lapse imaging, which would help more clearly delineate the emergence of pathologic processes and would establish a clear link between the identified phenotypes and temporal timeline of events.

## Materials and methods

### Cell medium composition for maintenance and assay

MCF10A cells were maintained in standard culture conditions, as previously described^17^. Growth medium was composed of DMEM/F12 (GIBCO), 5% horse serum (GIBCO), 20 ng/ml EGF (Sigma), 0.5ug/ml hydrocortisone (Sigma), 100 ng/ml cholera toxin (Sigma), 10ug/ml insulin (Sigma), and 1:100 100× Pen/Strep (GIBCO).The assay medium utilized in the course of the experiment was the same as the growth medium, with the exception that horse serum was at 2%, and that in some experimental conditions EGF was not added to the basal medium.

### PEG hydrogels

For convenience, we reproduce below methodological details originally available in refs^13,14^. Briefly, the factor XIIIa substrate peptides TG-MMP-Lys and TG-Gln were added to eight-arm PEG vinylsulphone (PEG-VS) in a 1.2-fold molar excess over VS groups in 0.3 M triethanolamine (pH 8.0) at 37 °C for 2 h. The reaction solution was subsequently dialysed (SlideA-Lyzer 7 K, MWCO or Snake Skin, MWCO 10 K, PIERCE) against ultrapure water for 3 days at 4 °C. After dialysis, the product (8-PEG–MMP-Lys and 8-PEG-Gln, respectively) was lyophilized to obtain a white powder.

### Factor XIII activation

Activation of factor XIII (FXIII) was achieved as described previously^14^. Briefly, 1 ml of 200U/ml FXIII (CSL Behring) was incubated for 30 min at 37 °C with 100 μl of 20U/ml thrombin (SigmaAldrich) in the presence of 2.5 mM CaCl_2_. Activated FXIII was aliquoted and stored at − 80 °C until use.

### HTS robotic mixing and dispensing

For convenience, we reproduce below methodological details originally available in ref.^13,26^. High throughput combinatorial screening of 3D microenvironments was performed as described using a Hamilton Microlab StarPlus automatic liquid handling robot with a Nanopipettor head. All automated steps were programmed with MicroLab Vector Software version 4.1.1 (HAMILTON Bonaduz AG). In brief, 8 combinations of PEG precursor solutions (comprising 2 MMP sensitivities × 4 stiffnesses (1, 1.5, 2, 3% wt/vol PEG, corresponding to 0.5 KPa, 2 KPa, 4 KPa, 8 KPa); MMP sensitivities were controlled by incorporating the two MMP substrates GPQG ↓IWGQ and VPMS ↓MRGG in the gel backbone) were mixed robotically and aliquoted into wells of a 384-well plate. ECM proteins were thawed on ice and dispensed (including blank control) into the gel precursor-filled wells to produce hydrogel precursors with 64 unique combinations of mechanical properties (MP), MMP sensitivities/degradability (DG) and ECM components (EC). ECM proteins were mixed to achieve a final concentration of 0.1 mg/ml. A 96-well plate was prepared with differentiation medium containing the soluble EGF (10 ng/ml) and a blank control.

MCF10A cells were trypsinized and resuspended at a concentration of 2 × 10^6^ cells/ml and kept on ice. Simultaneously, frozen aliquots of FXIIIa were thawed and also kept on ice. Cells were then dispensed into the wells containing gel precursors, followed by dispensing and mixing of FXIIIa. The final volume of each gel was 4 μl. These cell-containing hydrogel mixtures were then immediately dispensed into a 384-well plate, incubated for 20 min, followed by having media containing EGF or a blank control dispensed in a timed, automated manner.

### High-throughput imaging

For convenience, we reproduce below methodological details originally available in^13^. Plates were fixed with 4% paraformaldehyde 8 days after induction of reprogramming and then stained with Alexa647-conjugated Phalloidin (Life Technologies). Imaging was performed on a BD Pathway 435 automated imaging system (BD Biosciences). At every xy-position, that is, for every well, 6 images were captured across a z-stack height of 800 μm. For each well, these 6 images in each channel were collapsed into a single additive image.

All images were processed using algorithms developed in CellProfiler v.9777 (Broad Institute). Collapsed image stacks for each well in the AF647 channels were input. In an initial analysis, images were subjected to a threshold and segmented to obtain total colony numbers per well.

### Image classification using machine learning-based software

All colonies segmented in Cell Profiler were input into Cell Profiler Analyst v.11710. At least 30 individual samples from each colony were used as training sets, followed by validation on at least 10 images. This process was iterated until at least 15 images were correctly classified, using a Random Forest classifier.

### HTS data processing and statistical analysis

For convenience, we reproduce below methodological details originally available in^14^. Matlab R2010b (Mathworks) was used to process and visually explore the data. Data for each well were averaged over experimental triplicates to obtain averages, standard deviations and standard errors of the mean for each unique microenvironmental condition. The ‘number of phenotype positive colonies (count per well)’ output was selected from this data set and ranked. The top 5% of all conditions were extracted from this ranked set and their corresponding microenvironmental conditions were identified. Each microenvironment in these top conditions was represented by a set of 4 numbers corresponding to a category (MP, DG, EC, SF). For the identified top conditions, counts for each condition within each category were added, and all counts were represented as a percentage within each category.

To construct the generalized linear models, data were first input into R V2.14.2. GLM models, which took into account all possible interaction terms specified for analysis of number of GFP^+^ colonies. The step AIC procedure was run to obtain optimal models based on the Akaike criterion. The GLM procedure in SAS v9.0 software (SAS Institute) was used to compute a least squares mean value for each factor within every category, and differences of least-squares means ± standard errors with the control were tested for significance. The used models considered the effects of MP, DG, EC and SF, as well as interactions determined to be significant. For all parametric tests, normality of the residues and homogeneity of the variance were examined in QQ and Tukey–Anscombe plots, respectively. Principal component and singular value decomposition analyses were done with the svd function in R.
